# Visual Reassessment with Flux-Interval Plot Configuration after Automatic Classification for Accurate Atrial Fibrillation Detection by Photoplethysmography

**DOI:** 10.3390/diagnostics12061304

**Published:** 2022-05-24

**Authors:** Justin Chu, Wen-Tse Yang, Yao-Ting Chang, Fu-Liang Yang

**Affiliations:** 1 Research Center for Applied Sciences, Academia Sinica, 128 Academia Rd., Sec. 2, Nankang, Taipei City 115-29, Taiwan; nk95061313@gate.sinica.edu.tw (J.C.); d06631002@ntu.edu.tw (W.-T.Y.); 2 Department of Biomechatronics Engineering, National Taiwan University, No. 1, Sec. 4, Roosevelt Rd., Taipei City 10607, Taiwan; 3 Division of Cardiology, Department of Internal Medicine, Taipei Tzu Chi Hospital, Buddhist Tzu Chi Medical Foundation, No. 289, Jianguo Rd., Xindian Dist., New Taipei City 231-42, Taiwan

**Keywords:** atrial fibrillation, arrhythmia, photoplethysmography, blood flux, reassessment, configuration

## Abstract

Atrial fibrillation (AFib) is a common type of arrhythmia that is often clinically asymptomatic, which increases the risk of stroke significantly but can be prevented with anticoagulation. The photoplethysmogram (PPG) has recently attracted a lot of attention as a surrogate for electrocardiography (ECG) on atrial fibrillation (AFib) detection, with its out-of-hospital usability for rapid screening or long-term monitoring. Previous studies on AFib detection via PPG signals have achieved good results, but were short of intuitive criteria like ECG p-wave absence or not, especially while using interval randomness to detect AFib suffering from conjunction with premature contractions (PAC/PVC). In this study, we newly developed a PPG flux (pulse amplitude) and interval plots-based methodology, simply comprising an irregularity index threshold of 20 and regression error threshold of 0.06 for the precise automatic detection of AFib. The proposed method with automated detection on AFib shows a combined sensitivity, specificity, accuracy, and precision of 1, 0.995, 0.995, and 0.952 across the 460 samples. Furthermore, the flux-interval plot configuration also acts as a very intuitive tool for visual reassessment to confirm the automatic detection of AFib by its distinctive plot pattern compared to other cardiac rhythms. The study demonstrated that exclusive 2 false-positive cases could be corrected after the reassessment. With the methodology’s background theory well established, the detection process automated and visualized, and the PPG sensors already extensively used, this technology is very user-friendly and convincing for promoted to in-house AFib diagnostics.

## 1. Introduction

### 1.1. Background

Atrial fibrillation (AFib) is the main factor of cardioembolic stroke and is associated with a 3.7-fold increase in all-cause death [1]. AFib happens when the atrium depolarizes fast and irregularly, which leads to contractile dysfunction. However, adequate treatments are hindered for those patients with AFib due to as many as 50~87% of them being initially asymptomatic [2]. Thus, accurate and convenient automated AFib detection methods have always been a popular research topic for their demands and challenges.

The golden standard of AFib detection is conducted through analyzing 12-lead electrocardiogram (ECG) signals, which is inconvenient for large-scale screening programs and not suitable for continuous or long-term monitoring. While ECG-based methods require a minimum of two electrodes with stable contact with the skin, PPG-based methods only need a single point of contact. Just like ECG, the photoplethysmogram (PPG) signal also originates from the cardiac cycle, which inherits the ability to extract the same variability features but is more circumstantial, as it is the measurement of blood flow volume difference in the capillary caused by each heartbeat. A study on the extracted features between ECG and PPG was conducted to confirm the viability of using PPG to replace ECG for heart rate variability (HRV) features [3]. PPG signals also reflect one’s hemodynamic characteristics that contain information on cardiac activity, cardiovascular condition, sympathetic and parasympathetic nervous system interaction, and hemoglobin level from a peripheral site [4,5,6]. These characteristics could shed new light on different AFib detection methods by revealing new information, namely the change in every heartbeat’s stroke volume, that was not accessible through ECG. However, previous studies are fixated on only using interval-related features shared by both ECG and PPG.

**Figure 1:**
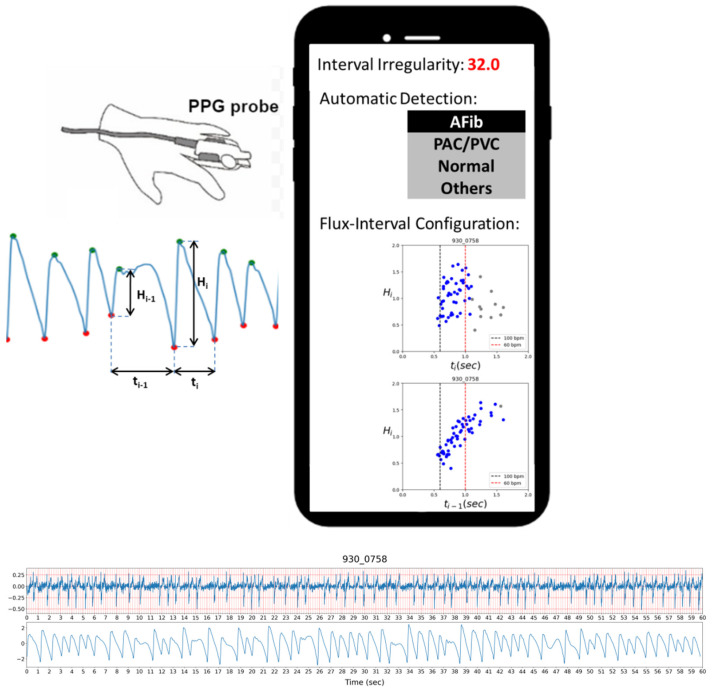
Demonstration of automatic AFib detection and visual assessment displayed on a smartphone with a PPG probe.

PPG sensors are more affordable, easier to use, and already commonly implemented on various wearable devices, making them a potentially convenient alternative for AFib detection [7]. A common application is to combine a PPG device with a mobile phone by either connecting the phone to a device with PPG sensors or utilizing the smartphone’s camera as the PPG sensor. A demonstration of a use case scenario with a smartphone using our methodology is depicted in Figure 1. The figure shows a concept of a mobile phone app presenting the users with information regarding the detection result of the analyzed PPG signal.

### 1.2. Previous Studies

A detailed review of previous studies was conducted by Pereira et al. on different approaches to PPG-based AFib detection [8]. In their paper, previous works are split into three groups by the approaches they use, which are statistical analysis approaches, machine learning approaches, and deep learning approaches. As Pereira et al. mentioned, previous PPG-based AFib detection methods suffer from many shortcomings and face many different challenges in different aspects, such as having difficulty in distinguishing AFib from other AFib like cardiac arrhythmia and over-complicated models for users to interpret the results.

In general, statistical analysis approaches extract RR interval (R peak to R peak) series features and spectral entropy and then try to find the best threshold values with ROC curves [9,10,11,12,13]. Simple statistical analysis methods are robust and intuitive but can be less effective compared to the more advanced machine learning and deep learning methods.

Due to the randomness of atrial contraction for AFib in combination with significant variability on inter-personal differences, the PPG signals can present in many different waveforms. Due to these immense differences, for machine learning methods to cover all the bases might require an immensely large amount of training data which can be very hard to come by. While machine learning methods offer more effective and optimized algorithms, much like the statistical analysis methods, their performance is still bound by the limitation of how good the features are that were available to the model.

To overcome this limitation, researchers turn to deep-learning approaches with automatic feature extraction like convolution neural network (CNN). Convolution neural networks are commonly used in solving image-related problems for their ability to extract important and representative patterns as features automatically through different filters from the data input [14]. Kwon et al. applied CNN models and mentioned previous algorithms that were mostly based on RR interval series and HRV-related features and had very little discussion on PPG signal amplitude [15]. A deep learning approach with CNN layers may allow the model to learn about the amplitude information, but due to the nature of being a black-box algorithm, it is hard to interpret or confirm how and whether the model actually uses the amplitude information.

Methods proposed in previous works may achieve promising performance but the explainability and transparency of the detection model were not very reassuring from the regular user’s point of view. Based on the work by Pereira et al., we further organized the previous studies into different groups by where their feature arise from each study used in Table 1 to categorize different approaches and show how underutilized the pulse amplitude information is.

### 1.3. Aim of This Study

When identifying AFib with ECG, the hallmark would be the irregularly irregular ventricular response, absence of discernable P-wave, or even the presence of chaotic fibrillatory F-wave. We seek to overcome the limitation of needing a minimum of two points of contact on ECG-based methods by solely relying on the PPG signal. Previous studies on using PPG for AFib detection require complex model and rely heavily on using traditional inter-pulse interval features from ECG that are also available on PPG signals, such as RR time series features and HRV features, while contraction-related information is often completely neglected.

Among the reviewed works, we took particular interest in the study by Schack et al., where they briefly mentioned the correlation between pulse wave height and diastolic length and achieved perfect detection of Afib, as we believe PPG amplitude holds the key information on further improving PPG-based AFib detection [32].

In this study, we aim to propose a method that gives an accurate and informative result by providing an easily understandable visual characterization of the physiological basis for every prediction result. This would help inform the users of the basis of the model’s decision to bolster user confidence and allow for a second opinion to double-check the result as previous works were able to give users a concise result, but users may find it obscure and out of touch. Here we have demonstrated a novel approach with simple and intuitive criteria that is easy and explainable based on the physiological characteristic of the cardiac cycle.

The presented model uses the automated AFib detection result with their PPG pulse amplitude and interval correlation altogether. AFib is known to reduce one’s cardia output with abnormal atrial contraction and diastolic dysfunction [5]. Since the PPG signal is related to blood flow across the peripheral circulation, the stroke volume is correlated to PPG signal amplitude with a strong correlation between each pulse pressure and its preceding diastole [33]. We expect our model to be able to accurately distinguish AFib from other cardiac arrhythmia and normal sinus rhythm (NSR) by analyzing one’s PPG signal amplitude changes which act as a surrogate of beat-to-beat stroke volume variation.

## 2. Material and Methods

In this study, 5264 samples of 1-min ECG and PPG signals were recorded from 2632 subjects. The study was started by recruiting participants in community-based health care centers. All subjects were fully informed and gave written consent for the recording and usage of data in this study. Samples with AFib are labeled from ECG as a reference to PPG signals. The study was approved by the Institutional Review Board of Academia Sinica, Taiwan (Application No: AS-IRB01-16081).

### 2.1. Measurement Protocol

The test subjects are asked to sit on the chair in a rest condition for at least 5 min for a questionnaire. Personal information with sex, age, smoking habit, familial history of disorders, height, weight, waist circumference, SpO_2_ (peripheral oxygen saturation), blood pressure, blood glucose, and HbA1C are asked or measured by commercial products listed in the next section. The subjects are then set up with ECG patches for lead I angle and PPG finger clips on index fingers for consecutive two 1-min recordings of waveform signals.

### 2.2. Hardware

The devices and instruments for the experiment are as follows: digiO2 POM-201 (digiO2, Miaoli, Taiwan) for SpO_2_. Omron HEM-7320 (Omron Healthcare, Kyoto, Japan) for blood pressure. Roche Accu-check mobile (Roche Diabetes Care, Indianapolis, IN, USA) for blood glucose. SEIMENS DCA Vantage Analyzer (Siemens Medical Solutions Diagnostics, Tarrytown, NY, USA) for HbA1C. CardioChek PA analyzer (Polymer Technology Systems, Indianapolis, IN, USA) for blood lipid. The signal of PPG is recorded from TI (Texas Instruments, Dallas, TX, USA) AFE4490 module and ECG from ADI (Analog Devices Inc., Norwood, MA, USA) AD8232-EVALZ.

### 2.3. Data Preprocess

After basic filtering of 0.1~40 Hz, automated valley and peak detection on PPG signals were conducted. Python 3 modules peakutils (ver. 1.1.1) were applied for the valley and peak detection. Peak detection was applied and cross-validated with two corresponding neighboring valleys. Valleys were also validated with continuous positive slopes. Based on the valleys, the PPG signal is broken into segments of singular pulses for later use.

Each sample’s cardiac rhythm was labeled based on its ECG signals accordingly by corresponding author Y.T. Chang, who serves as a cardiology specialist and clinical electrophysiologist. The labeled samples were selected from all samples but with interval irregularity index (*III*, see Method section) above 10. In addition, we randomly selected 245 samples with *III* under 10 to balance the distribution and to approximate the 10:1 ratio of non-AFib vs. AFib. The results labeled as ground truth are 286 NSR, 73 PAC (premature atrial contraction), 59 PVC (premature ventricle contraction), 40 AFib, and 2 atrioventricular blocks attributed to other types of arrhythmic rhythms.

### 2.4. Extraction of Irregularity and Regression RMSE of Main Cluster

**Figure 2:**
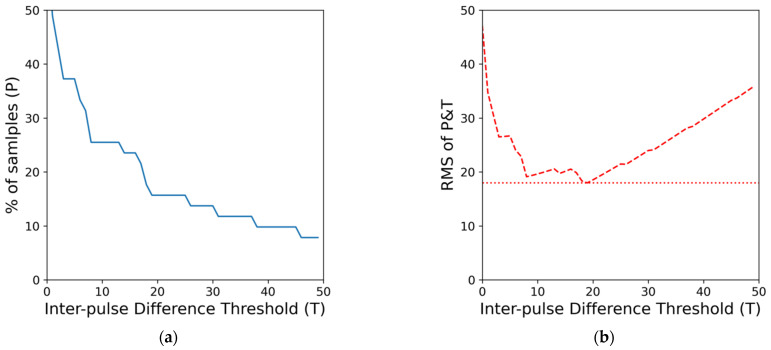
An example of H-index inspired interval irregularity index (*III*) calculation. The resulted *III* is 18.0. (**a**) Shows the % of samples satisfying different threshold values. (**b**) Shows the quadratic mean of P&T across different thresholds and where the minimum value is located.

First, an H-index inspired index is designed to represent the irregularity of each sample set and be used to determine if the signal segment contains enough possible AFib pulses. The index is calculated by finding the smallest quadratic mean (root mean square) of the thresholds of percentage difference of consecutive pulse interval (*T*) and the proportion of pulse satisfied said interval threshold (*P*) as summarized in Equation (1). While previous studies have often used RR time interval features in an absolute time difference of milliseconds, we opt to use the relative difference in % when it comes to inter-pulse difference. We believe the change in pulse length should take the specific person’s current heart rate into account. A 10-millisecond change in pulse between a person with a heart rate of 60 beats/minute and another person with a heart rate of 80 beats/minute does have a significant difference. Thus we use the relative difference in percentage instead of using an absolute value shared across different samples. This resulting value will be the interval irregularity index for the minute-long signal sample and be denoted as *III*. The process of finding the irregularity is also illustrated in Figure 2.

(1) $$III=min{\left\{\;\sqrt{\frac{T_{i}{}^{2}+P_{i}{}^{2}}{2}}\;\right\}}_{i=1}^{50}$$

**Figure 3:**
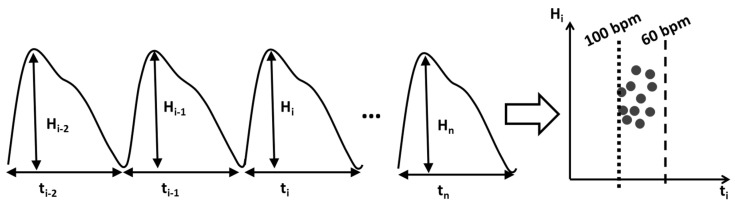
Illustration of the transformation of PPG signals to flux-interval plot with *H_i_* vs. *t_i_*.

Then, for each pulse segmented by valley detection, the sequences of pulse amplitudes (*H_i_*) were normalized by dividing individual pulse amplitude by its average and then paired together with intervals with different offsets. For each sample, the sets of (*t_i−_*_1_, *H_i_*) and (*t_i_*, *H_i_*) were then visualized into a scatter plot as shown in Figure 3 (later referred to as flux-interval plot) to form the basis of our method. From the flux-interval plot, we can observe that NSR, PAC, PVC, and AFib samples display distinctively different patterns. Flux-interval plot allows users to assess their changes in cardio-output over time and identify different cardiac rhythms with their distinctive patterns, much like identifying AFib from ECG using only a few iconic criteria. We expect any well-informed user can easily differentiate normal and abnormal rhythms easily based on the unique patterns without needing much training.

**Figure 4:**
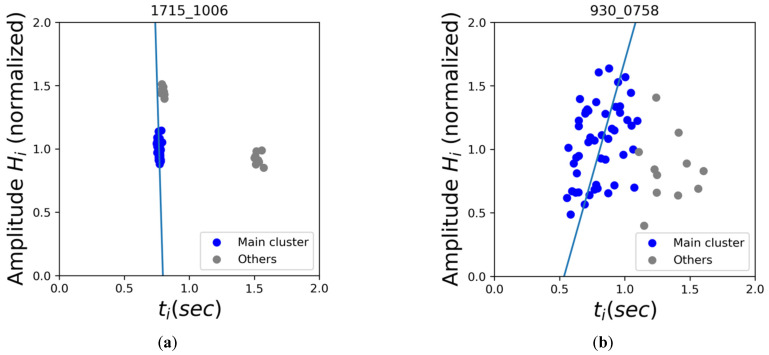
Examples of orthogonal regression line on the main cluster of (**a**) PVC sample and (**b**) AFib sample.

On each sample set of (*t_i_*_−1_, *H_i_*) and (*t_i_*, *H_i_*), DBSCAN (Density-based spatial clustering of applications with noise) clustering was applied. Conceptually the clustering result would reflect the types of pulses within the signal based on each pulse’s location flux-interval plot. The orthogonal regression was then applied to the main cluster (the cluster with the most data) of (*t_i_*, *H_i_*) sets, and the residual errors were recorded in the form of root-mean-squared error (RMSE). As the examples show in Figure 4, we expected that the fitted regression line of the main cluster would result in a smaller RMSE on premature contractions as they tend to have tight clusters as opposed to the more scattered distribution of AFib on the flux-interval plot. We intend to use this denoted “Regression RMSE of Main Cluster” as a simple index to represent the degree of dispersion of each sample.

### 2.5. Hemodynamic Model and (t_i−__*1*_, H_i_)

Previous studies, such as the analysis of cardiac arrhythmia by Pfeiffer et al., have indicated a strong correlation between (*t_i__−_*_1_, *H_i_*), but the reasoning behind this correlation was not clearly explained [33]. Here we try to explain the hemodynamic cause of (*t_i__−_*_1_, *H_i_*) correlation in detail as below, and our algorithm would also use cardiac electrophysiology to enhance the discrimination power.

A person’s stroke volume is determined by how much blood is ejected during contraction. As shown in Equation (2), there are three factors affecting one’s stroke volume (or flux), namely preload, contractility, and afterload. During the diastole of the heart, the blood accumulates in the ventricle before the ventricular contraction, and the end-diastolic pressure is so-called preload. The normal diastole starts with rapid filling due to passive ventricular suction and follows with active filling by atrial contraction. While ejecting the blood from the heart, it has to overcome the systematic arterial pressure that is pushing back against the aortic valve, which is referred to as afterload.

(2) Stroke volume=f(Preload, Contractility, Afterload)

For simplicity, it is assumed that for each person within each one-minute measurement, their contractility and afterload should remain largely the same, thus, they are treated as constant here. Therefore we substitute the contractility and afterload with a Constant to transform the model function into Equation (3).

(3) $$\mathrm{Stroke}\;\mathrm{volume}=f_{p}\left(\mathrm{Preload},\;\mathrm{Constant}\right)$$

During the cardiac cycles, the preload period for pulse *i* is represented by the preceding pulse’s interval ($t_{i-1}$). In the early passive diastolic phase, the atrium works as a reservoir of blood and the filling volume is related to the ventricular suction pressure and the filling time. Since the preceding pulse interval ($t_{i-1}$) would largely affect the filling time and the flow rate is in proportion to ventricular suction pressure. The integral of these 2 items may represent the preload, which like an hourglass between heart contractions, we acquired Equation (4).

(4) $$\mathrm{Stroke}\;\mathrm{volume}=f_{p}\left(flow\;rate\;\left(s\right)\times t_{i-1},\;\right)+\mathrm{active}\;\mathrm{filling}$$

Since the atrial mechanical function is impaired in AFib, the active diastolic filling is negligible in AFib. This simplified the model into Equation (5) and allows us to focus on how the preload period affects one’s stroke volume.

(5) $$\mathrm{Stroke}\;\mathrm{volume}=f_{p}\left(flow\;rate\;\left(s\right)\times t_{i-1}\;\right)$$

PPG amplitude is in proportion to stroke volume, but their relationship has yet to be properly modeled. The PPG signal amplitude is correlated to the amount of blood flow but is hard to model due to many other different variables such as systematic vascular resistance. These various variables can result in inter- and intra-personal differences when comparing. In this study, we assume that within each one-minute PPG measurement, the intra-personal difference on variables other than stroke volume remains largely the same and thus can be neglected. Based on this assumption, we can interchange the stroke volume with PPG amplitude and result in Equation (6).

(6) $$\mathrm{Amplitude}\;\left(H_{i}\right)=flow\;rate\;\left(s\right)\times t_{i-1}$$

This explains why the flux-interval plot of *H_i_* vs. *t_i−_*_1_ would present with a positive slope, especially in patients with AFib.

### 2.6. Electrophysiology Model and (t_i_, H_i_)

The AFib is well-known as chaotic heart rhythm, and previous studies that have aimed to evaluate the randomness of AFib found the auto-correlation between each RR interval was low [34]. However, this relationship would not be random in sinus rhythm, PAC, or PVC.

The coupling interval, namely the RR-interval preceding the premature beats (*t_i__−_*_1_), is traditionally believed to be constant in a stable sinus cycle length [35]. In addition, the ECG morphology of a premature beat has a fixed relationship with its coupling interval. It is because the firing of a PAC or PVC is originated from the same piece of the myocardium with the same mechanism. Although various kinds of premature beats may present in a patient, a dominant morphology with its fixed coupling interval would be observed more frequently.

The relationship of returning cycles, namely the RR-interval following the premature beats (*t_i_*), is also not random [36]. When a PAC fires with a shortened RR-interval (*t_i__−_*_1_), returning cycle would be prolonged if the sinoatrial node (SA node) is not electrical penetrated by the electrical wave of PAC. The electrical wavefronts of PAC and the SA node collide somewhere in the atrium, and the returning cycle would compensate for the short coupling interval, and the summation of the coupling interval and the returning cycle would equal to two times of sinus cycle length. Even when a PAC with a further shorter RR-interval is encountered, the electrical wavefront will penetrate and reset the SA, node, and the return cycle would be nearly the same as the basic sinus cycle length. Since the PAC falls in the last 60~80% of the basic sinus cycle would fall in the above conditions, the length would not be random and depend on the characteristics of the SA node and PAC coupling interval (*t_i__−_*_1_) This condition remains similar for PVC, since its electrical wave must penetrate the atrioventricular node (AV node) first and requires a longer conduction time before reaching the SA node.

We suggested that coupling interval (*t_i__−_*_1_) and returning cycle (*t_i_*) would present a stable relationship in patients with PAC or PVC. Since the flux (*H_i_*) is associated with t_i-1_ explained in the hemodynamic section, and the coupling interval is presented with constant more frequently, the flux-interval plot of returning cycle (*t_i_*, *H_i_*) would be non-random and present with a few clusters of points. On the contrary, although the flux-interval plot of (*t_i__−_*_1_, *H_i_*) presents with a positive linear slope in AFib, the flux-interval plot of (*t_i_*, *H_i_*) would be dispersed because of the low correlation of *t_i__−_*_1_ and *t_i_* is low in AFib.

## 3. Results

**Figure 5:**
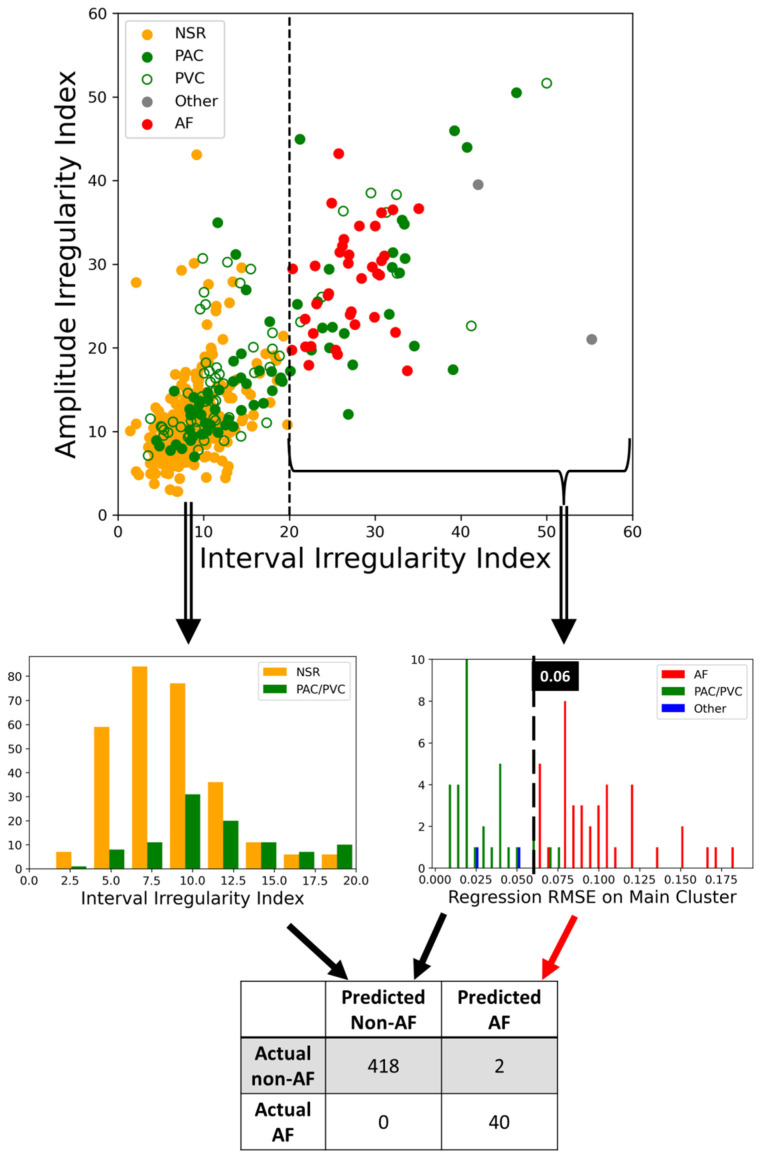
Irregularity distribution of different types of heartbeat rhythm and automatic detection process of separating AFib from different arrhythmic rhythm with irregularity and RMSE of orthogonal regression on the main cluster from (*t_i_*, *H_i_*) relation.

Based on our data set, we found that with two simple conditions of checking irregularity and the regression RMSE of the main cluster, we can correctly determine if the 60 s PPG segments contain AFib with only two false positives without any false negatives. In Figure 5, we visualized how each condition separates different sinus rhythms in a tree-like structure. The same steps of acquiring interval irregularity were adopted on amplitude here for the purpose of matching the scale of the X and Y-axis. From the top plot, we can clearly see AFib samples (red dots) have significantly larger irregularities compared to the others and with some premature contractions data in the mix. With an interval irregularity of 20 as the boundary, all NSR and AFib could be separated perfectly. Despite being labeled as premature contractions, the data with irregularity under 20 are generally NSR with occasional premature contractions which are considered normal and mostly harmless. On the other hand, the premature contraction data with irregularity over 20, these PAC and PVC usually have a bigeminy, trigeminy, or even quadrigeminy rhythm, which leads to two or more distinctive clusters each with tight distribution on their flux-interval plot. Based on the features previous studies used, differentiating these PAC and PVC with large irregularity from AFib is what their methods may struggle on. On the right subplot of Figure 5, we can see that by assessing the orthogonal regression RMSE of the main cluster, we can convincingly pick out the AFib data from the pile of data that had irregularity over 20 with regression RMSE of the main cluster at 0.06 as the boundary. Based on these two simple yet powerful threshold conditions that require no complex computation we can automate the classification of AFib with the performance of 1, 0.995, 0.995, and 0.952 for sensitivity, specificity, accuracy, and precision, respectively. This result suggests our model is not inferior to the majority of the 24 studies reviewed by Pereira et al.

**Figure 6:**
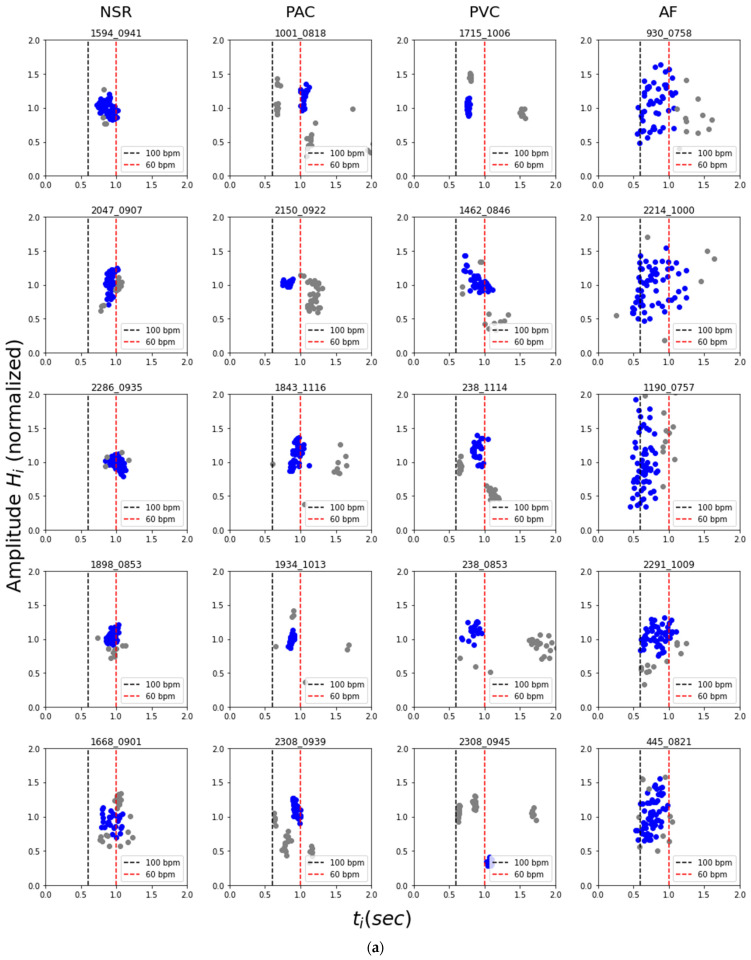

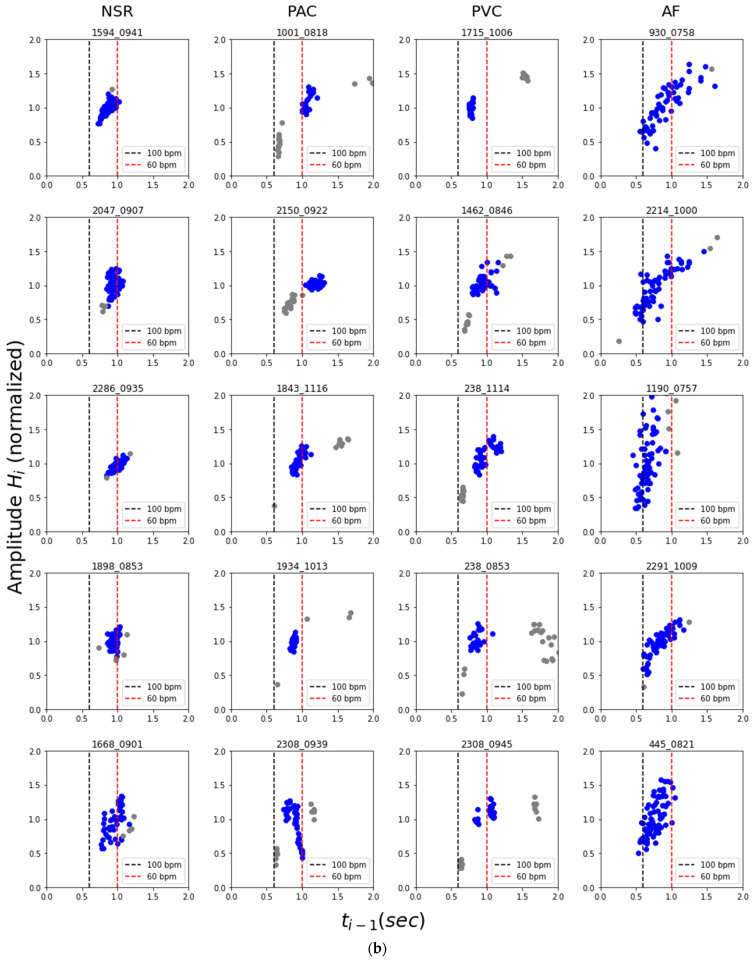
Typical pattern of the flux-interval plot for different cardiac rhythms. Blue dots represent the main cluster for each sample in the plot. (**a**) Flux-interval plot in the configuration of *H_i_* vs. *t_i_*, (**b**) flux-interval plot in the configuration of *H_i_* vs. *t_i−_*_1_.

Figure 6a,b demonstrated how the (*t_i__−_*_1_, *H_i_*), (*t_i_*, *H_i_*) relations generally manifest themselves for different types of cardiac rhythms on the flux-interval plot. The most iconic and representative characteristics are summarized in Table 2 as a guideline for differentiating types of cardiac rhythms. NSR would mostly be in a single tight cluster with little variation both in terms of interval and amplitude. PAC and PVC with rhythmic premature contractions would often present themselves as two or more distinct cluster of premature beats, normal beats, and the extended normal beat right after each premature beat. For samples of NSR, PAC, and PVC, their style of data distribution would appear consistent on both (*t_i__−_*_1_, *H_i_*) and (*t_i_*, *H_i_*) flux-interval plots. On the other hand, the AFib pattern would often look like a semi-tight scatter of points forming a positive slope on the (*t_i__−_*_1_, *H_i_*) plot and a widely scattered distribution on the (*t_i_*, *H_i_*) plot. This visualization helps us understand the fundamental difference between types of cardiac rhythms for classification. The observed distinct characteristic of different rhythms on the flux-interval plot is consistent with our hypothesis on identifying cardiac rhythms based on similar characteristics when identifying them via ECG. The plots cluster pattern correlates to the functionality of the atrium and the irregularity reflects the rhythmicity. Though with the cluster pattern, we can differentiate AFib from PAC and PVC, however, it appears that PAC and PVC are indistinguishable on the flux-interval plot.

## 4. Discussion

**Figure 7:**
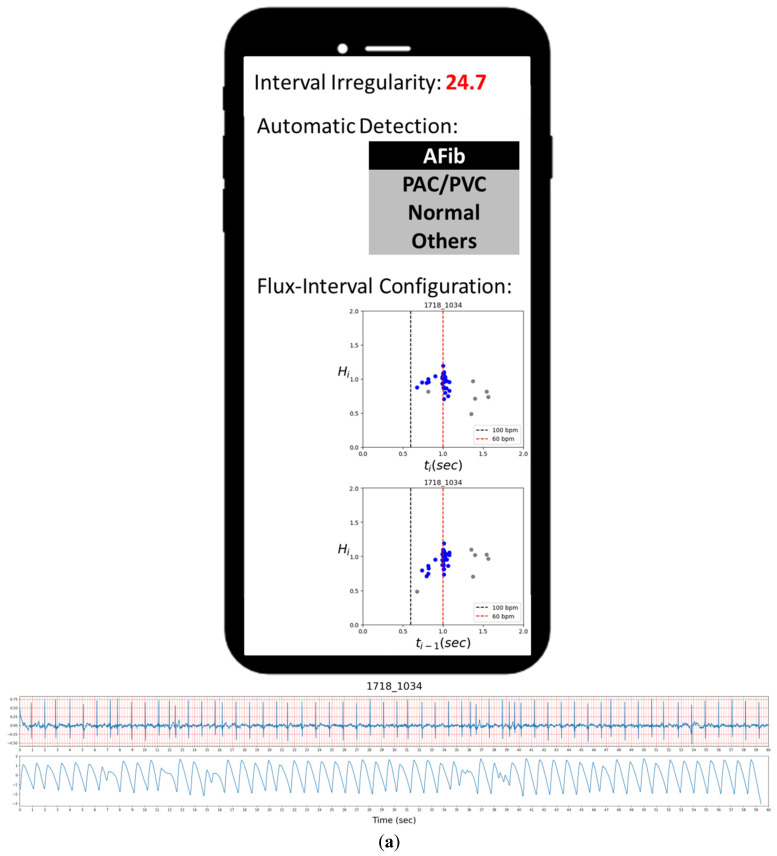

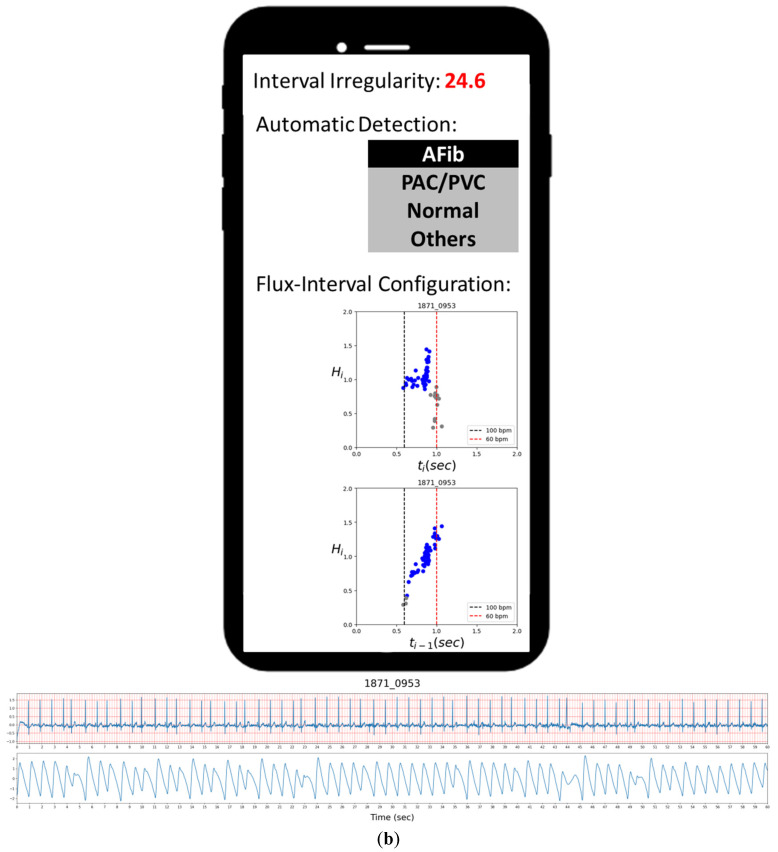
Two false-positive cases of premature contractions (non-AFib) classified as AFib. In both cases, frequent PACs were encountered, but these PACs came with different coupling intervals if measured carefully (**a**,**b**). In addition to the PAC attacked in case (**a**), a short-run atrial tachycardia (AT) attacked around time mark 39 s.

Compared to previous studies, our approach is more in line with the statistical analysis with assistance from machine learning methods while focusing heavily on deriving meaningful physiological features via novel approaches. While many previous works already achieve perfect or near-perfect results through different algorithms, their behind the scene processes are hidden to the users, like a black box. With our innovative approach with the flux-interval plot, we can provide a visual presentation on why and how we can effectively and convincingly distinguish AFib from other types of cardiac arrhythmia. While introducing a novel predictive analytic model to new users, their question on its usability often resides in how accurate it is and how they can trust the result. Our straightforward model offers good accuracy on par with the best performing studies, while added intuitive visual presentation allows the user to carry out a “trust, but verify” approach. The extra information grants users access to a more informative result and the ability to double-check the basis of each prediction with their own eyes for increasing use case confidence. This has the potential to bring significant improvement and contribute to new functionality to the PPG-based AFib detection method in the future. While our method produces accurate automated prediction via simple parameters, there still are 2 false-positive cases of non-AFib arrhythmia classified as Afib, as presented in Figure 7.

### 4.1. Visual Reassessment

Figure 7a,b presents the two false-positive cases of ECG-labeled PAC samples misclassified as AFib by our automated detection, while their flux-interval plot tells a different story. Upon visually reassessing the flux-interval plots in both configurations following the guideline in Table 2, we can clearly see that in both cases, the (*t_i_*, *H_i_*) plot did not present itself in a single widely scattered cluster and multiple clusters can be identified. In the case of Figure 7a, it appears as sinus rhythms with PAC, but with a short episode of atrial tachycardia (multiple APC in short succession) in the mix at around 39 s mark, thus resulting in the resemblance of AFib. In the case of Figure 7b, the false-positive lies in the presence of various kinds of PAC with different coupling intervals (*t_i__−_*_1_). These false-positive results could be attributed to the limitation of the clustering algorithm to differentiate these turbulences of RR-interval when complex arrhythmias happen. Even when reading ECG signals, these complex arrhythmias are frequently confusing and require a clear ECG P-wave to figure out the answer in clinical practice.

**Figure 8:**
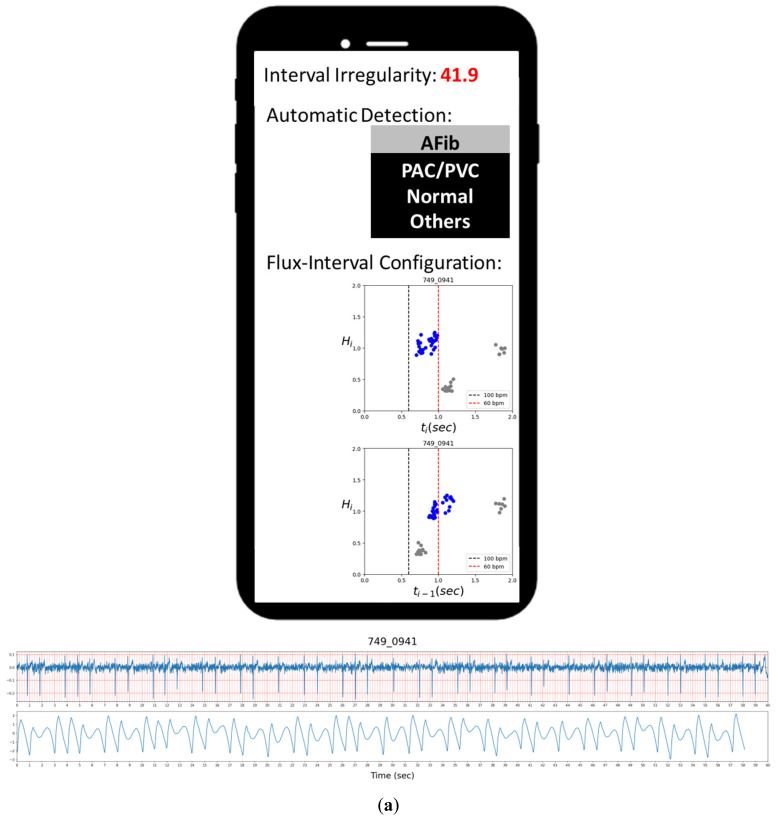

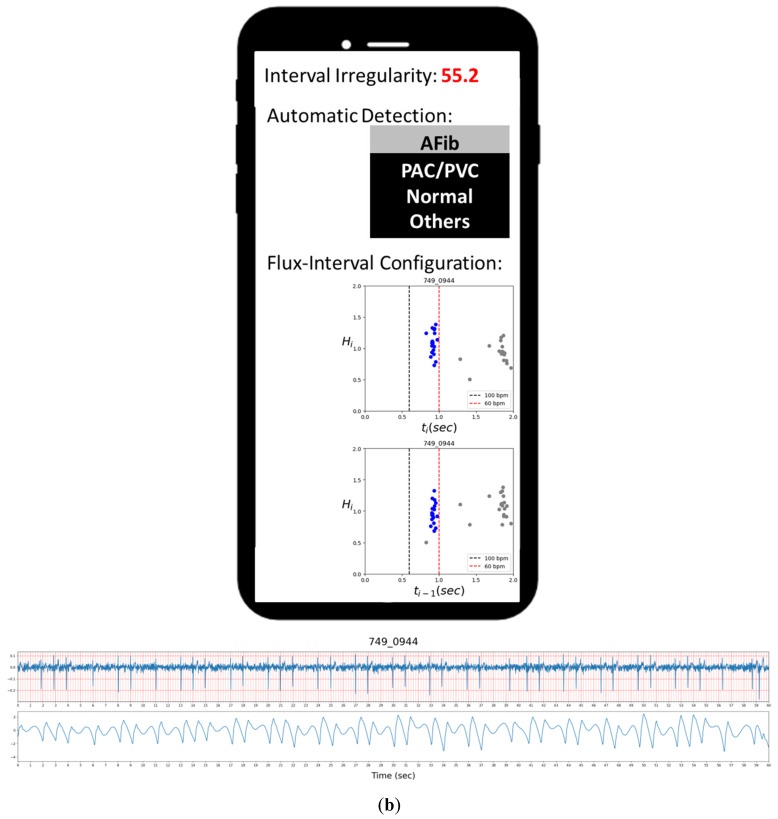
(**a**,**b**) Two cases of non-AFib (ECG labeled as others, atrioventricular block) correctly detected. In both cases, their irregularity indexes are very high, but their regression RMSE on the main cluster is less than the threshold of 0.06. Thus, they are automatically detected as non-AFib. Their flux interval configurations are similar to PAC/PVC but far from AFib.

In Figure 8, we show the two cases of atrioventricular block labeled as others, which are automatically detected as non-AFib. In both cases, their irregularity indexes are very high, but their regression RMSE on the main cluster is less than the threshold of 0.06. Thus, they are correctly detected as non-AFib. From looking at their flux-interval configurations following the guideline in Table 2, they are very similar to PAC/PVC but far from AFib. This suggests that even other types of arrhythmias may have similar characteristics with PAC/PVC on the flux-interval plots and can too be easily differentiated from AFib. The two cases demonstrated reassessment with flux-interval configuration is very useful for users to confirm automatic classification results.

### 4.2. Sample Size and Applicable Population

The participants were recruited in multiple community-based health care centers and aimed to exploit the potential utilization of PPG in blood chemistry tests and other physiology signals. Thus, the baseline epidemiologic questionnaire did not investigate the history of detailed disease entities, such as valvular heart disease. At first glance, the resulted sample size of 460 would appear to be insufficient for meaningful statistical analysis. This is due to the majority of 5264 samples being initially categorized as NSR for having very little inter-pulse interval changes. In another words, these samples’ pulse intervals are so consistent it is not possible for them to contain any AFib episode or any arrhythmic rhythm that could possibly be mistaken as AFib. We believe our survey is reasonable to represent a general population with our 5264 samples of 60 s ECG strips from 2632 volunteered subjects, which comprise a total of 87.7 h of recording. The overall time length of our data set may be significantly shorter compared to studies with 24 h Holter monitoring, but our dataset has covered a significant number of unique individuals. Regarding the prevalence of arrhythmia, it is difficult to compare our incidence with previous works directly as the population registry for disease prevalence was more commonly surveyed by medical database or 24-h Holter study [37]. In comparison, our data set includes 73 premature atrial complexes (73/5264, 1.4%), 59 premature ventricular complexes (59/5264, 1.1%), and 40 atrial fibrillations (40/5264, 0.8%). These numbers suggest the prevalence of arrhythmia in our dataset is not too far off from previous works.

As aforementioned, this study is based on a general population and intended for screening purposes and in-house AFib diagnostics. Our study was not designed for complex arrhythmia conditions or detecting heart diseases. For example, in the presence of atrial fibrillation and AV block at the same time, we might possibly not detect the condition and make the correct diagnosis, although this kind of more complex condition would be more likely to be encountered in a hospital setting. In addition, the mechanism of paroxysmal atrial fibrillation involves trigger activity, which is the same mechanism of the atrial premature complex. Thus, in the presence of multiple APC triggers and atrial tachycardia with variable conduction, it is also difficult to differentiate with the PPG signal only and it might sometimes even be misleading if only a short ECG strip was used. Fundamentally, our flux-interval plot is a personalized heart performance monitoring technique that keeps track of the relative changes in blood flux that is sensitive to sudden changes in heartbeat interval and blood flux. Further studies would be required on how specific heart disease would affect the model performance, but we expect this methodology to remain effective in AFib detection.

## 5. Conclusions

PPG signals provided blood flux information previously unavailable through ECG had shed new light on new methods for detecting atrial fibrillation and other arrhythmia patterns. In this study, we demonstrated how monitoring one’s change in blood flux (represented by PPG pulse amplitude) across a period of time can be an easy and effective way of detecting if the subject is having an atrial fibrillation episode. We found when projecting the PPG waveform data to the flux-interval plot, based on explainable physiology relation between pulse interval and amplitude, AFib could be easily characterized and convincingly differentiated from other arrhythmic rhythms. Previous AFib detection usually suffers from conjunction with PAC/PVC by PPG if only counting on interval randomness has been solved either through the automatic detection process or visual reassessment with this novel technology. While our proposed PPG method offers significant benefits over previous methods, like any other PPG-based method, it is still limited by the quality of the PPG signal and may require longer signal strips. The proposed method with automated detection on AFib shows a combined sensitivity, specificity, accuracy, and precision of 1, 0.995, 0.995, and 0.952 across 460 samples studied. Due to the generalized population sample of this study, studies focused on populations with some common types of heart disease could further validate the robustness and applicability of the methodology.

## Figures and Tables

**Table 1:** Features and methodology used in previous studies compare to this work.

Input Feature Type	Reference Work	Approach	Classification Methodology	Performance (Accuracy)
Time domain	[9,10,12,16]	Statistical analysis	Feature thresholds, logistic models	0.952–0.9645
	[17]	Machine learning	Support vector machines	0.9385
	[18]	Deep learning	Deep neural network	na
Time + Frequency domain	[3,11,13,19,20,21,22]	Statistical analysis	Feature thresholds, logistic models	0.91–0.981
	[23,24,25]	Machine learning	Decision tree, Support vector machines	0.95–0.957
CNN	[15,26,27,28,29,30]	Deep learning	Variations of CNN based deep neural network	0.95–0.999
CNN + Time + Frequency domain	[31]	Statistical analysis	Logistic model	0.918
Time + Frequency domain + pulse Amplitude	[32]	Machine learning	Support vector machines	1
Pulse interval + Pulse Amplitude	This Work	Statistical analysis	Feature thresholds, plot configuration	0.995

**Table 2:** Distinct flux-interval plot characteristics for visual reassessment.

	*H_i_* vs. *t_i__−_*_1_	*H_i_* vs. *t_i_*	Data Distribution
**AFib**	-Positive linear correlation -Pulses interval often exceed 0.6 s (100 bpm)	-Pulses interval often exceed 0.6 s (100 bpm)	-Single cluster with wide variation on both flux and interval
**PAC/PVC**	-Typically with more vertical pattern and multiple clusters	-Typically with more vertical pattern and multiple clusters	-low variation within individual cluster
**NSR**	-Compact pattern	-Compact pattern	-Single cluster with small variation on interval

## Data Availability

The data presented in this study are available on request to the corresponding author, but follows the aforementioned IRB guidelines.
